# Why specialize? An exploration of how clinical experience influences students’ motivation for specialist nursing education

**DOI:** 10.1186/s12912-026-05482-2

**Published:** 2026-10-08

**Authors:** Helena Kullenberg, Lena Skoglund, Maria Christidis

**Affiliations:** 1 Department of Health Promoting Science, Sophiahemmet University, Box 5606, 114 81 Stockholm, Sweden; 2 Department of Nursing Science, Sophiahemmet University, Stockholm, Sweden; 3 Department of Neurobiology, Care Sciences, and Society, Karolinska Institutet, Stockholm, Sweden

**Keywords:** From novice to expert, Higher education pedagogy, Motivation, Postgraduate education, Specialist nursing students, Situated learning

## Abstract

**Background:**

Existing research has examined motivation among specialist nursing students and identified factors influencing their pursuit of postgraduate education, including the role of clinical experience. However, it remains unclear whether and how motivation varies with years of clinical experience, and how such differences can be understood from a pedagogical perspective in postgraduate nursing education.

**Aim:**

To explore how nurses with different lengths of clinical experience articulate their motivations for pursuing specialist nursing education.

**Methods:**

This study was part of a larger survey examining the effects of clinical experience on self-efficacy, nursing competence, academic performance, and study outcomes among specialist nursing students. This paper presents a qualitative analysis of students’ reported motivation to pursue further education, based on qualitative free-text responses. The study used inductive descriptive thematic analysis, supplemented by cross-group comparisons and descriptive quantification of codes to identify patterns across clinical experience length. When interpreting the analysis, the study used Situated Learning theory and the Novice to Expert framework.

**Results:**

A total of 94 students participated. The analysis identified four overarching motivational themes: Professional Role Advancement, Expanded Participation, Contributing to the Improvement of Clinical Practice, and Personal Advantages. Overall, participants described a broad range of motivations for undertaking specialist nursing education, with emphasis across themes varying by group according to length of clinical experience.

**Conclusion:**

The findings suggest that length of prior clinical experience may shape not only what students know but also what they seek from formal education and how they engage with new knowledge. Consistent with perspectives on situated learning and the development of nursing expertise, the findings suggest that students’ prior experiences and positions in clinical practice may influence how they approach and make sense of further education. For educators, this underscores the importance of not assuming that all students have the same personal goals. Rather, education may serve different purposes for different students, and recognizing these varied orientations may help shape educational environments that respond to students’ different starting points and expectations while maintaining shared academic and professional goals.

**Supplementary Information:**

The online version contains supplementary material available at https://doi.org/10.1186/s12912-026-05482-2.

## Introduction

Modern healthcare systems are undergoing significant transformation driven by demographic shifts, multimorbidity, technological advances, and rising healthcare demands [1]. These changes increase the complexity of patient care and healthcare delivery, requiring nurses to manage intricate clinical situations and coordinated care needs [2]. As a result, specialist nursing competence has become vital to workforce sustainability, quality of care, and patient safety [3, 4].

A specialist nurse is a registered nurse who has advanced beyond basic nursing practice through additional education, clinical experience, and greater responsibilities [5]. Specialist nurses possess advanced clinical skills in a specific practice area, enabling them to assess complex care needs, deliver specialized treatment, and support patient and staff education [5]. In this context, specialist nursing education entails more than academic advancement. It prepares nurses for broader roles that address future healthcare challenges and shift toward more autonomous, specialized professional positions [6].

Against this backdrop, students entering specialist nursing educational programs are typically registered nurses with prior clinical experience, and their professional backgrounds can shape their motivations for further study [7]. Unlike many other groups in higher education, they are seasoned professionals already engaged in clinical practice. They bring established professional identities, experiential knowledge, and practice-based reasoning skills [8]. Because specialist nursing education is most often undertaken alongside clinical work, participation and completion may depend more on individual engagement than in undergraduate education [9, 10]. In addition, postgraduate nursing education expectations extend beyond knowledge acquisition to include advanced clinical judgment, care coordination, and practice development, which requires students to take an active role in their learning [11]. This suggests that motivation could be a particularly relevant factor to consider.

Motivation, broadly defined as the processes that initiate, direct, and sustain individuals’ efforts toward achieving goals, is widely acknowledged as important for engagement and learning in higher education [12, 13]. Motivation influences not only whether individuals choose to engage in a particular activity but also the intensity and persistence of that engagement over time. Researchers have commonly examined motivation in relation to individual psychological processes, such as interests, values, self-efficacy, and goal orientations, and highlighted the importance of contextual factors in how motivation is experienced and expressed [14, 15]. For nurses pursuing specialist education, motivation may therefore be understood in relation to both individual aspirations and the professional contexts in which they make educational decisions. Their motivations for further education may be connected to experiences gained through clinical practice, current professional roles, and considerations about future careers [16]. Consequently, a perspective that embeds learning and professional development within practice may offer particular value for understanding how motivations for specialist education are articulated and experienced.

### Previous research

In nursing education, researchers have primarily investigated motivation among undergraduate students and its relationship to engagement with studies and academic learning [17–19]. In undergraduate nursing education, motivation has been linked to intrinsic interest, autonomy-supportive teaching, study effort, and perceived learning outcomes [20], as well as engagement and persistence [18]. However, it may be important to distinguish between motivation to learn within an undergraduate educational program and motivation to pursue further education as a professional. Recent research has begun to address the latter among nurses undertaking postgraduate and specialist education. Studies have identified a range of personal, professional, and contextual factors that influence nurses’ decisions to pursue specialist education and their choice of specialty [21]. In a Swedish survey of 227 specialist nursing students, frequently reported incentives included opportunities for new tasks and responsibilities, intellectual challenges, and higher wages. Almost half of the students also identified financial benefits during education as an incentive to begin specialist studies, suggesting that the decision to undertake specialist education may be influenced by both professional aspirations and employment-related conditions [21]. Similarly, specialist nursing students’ decision-making motives have been qualitatively described in relation to professional development and career-related considerations [7]. A recent phenomenological study of registered nurses pursuing Master of Nursing Specialist education also examined participants’ motivations for undertaking specialist education in relation to their professional experiences [22]. The study identified motivations related not only to personal growth and career development, but also to workplace stress, perceived career-development dilemmas, and pursuing an academic degree. These findings suggest that postgraduate education may be pursued partly in response to dissatisfaction with current working conditions or limited career opportunities, rather than solely to acquire specialist knowledge [22].

Oldland et al. [23], who studied nurses undertaking postgraduate critical care education, found that motivations most often concerned acquiring knowledge and technical skills but also included desired personal and professional outcomes and behaviors. The findings suggested gaps between students’ desired learning outcomes and broader professional expectations, particularly around reflective practice, teamwork and communication, person-centered care, research skills, and clinical leadership. Similarly, Ingram et al. [24] identified promotion, personal growth, and the development of critical thinking and mastery of a chosen field as important motivations, while also highlighting challenges related to work–life balance, limited support, and financial strain. Together, these findings suggest that nurses’ motivations for postgraduate education are multifaceted and may encompass educational, professional, personal, and practical considerations.

In parallel, researchers have also examined postgraduate nursing students’ motivation in relation to learning processes during their education. For example, Bingen et al. [25] explored students’ motivation for learning and changes in academic motivation over postgraduate studies. Their study found that academic motivation changed over the course of the educational program, highlighting that motivation for learning is not necessarily stable throughout the educational process.

Taken together, previous research shows that nurses may pursue specialist and postgraduate education for diverse reasons, ranging from acquiring knowledge and developing professional competence to seeking career opportunities, improved working conditions, personal growth, or other practical benefits. However, less is known about how nurses’ prior clinical experience relates to their understanding and articulation of these motivations. Although Zhu et al. [22] explored nurses’ motivations in relation to professional experience, the study did not examine how nurses with different lengths of clinical experience may orient themselves towards specialist education. Similarly, Tiliander et al. [7, 21] identified factors influencing educational decisions and specialty choice, but did not examine how these motivations may relate to nurses’ accumulated clinical experience. Furthermore, Bingen et al. [25] focused on motivation within ongoing education rather than on how prior clinical experience may shape motivations for undertaking specialist education.

### Theoretical framework

This study draws on Situated Learning [26] and the From Novice to Expert framework [27]. These frameworks offer complementary interpretive perspectives for understanding how specialist nursing students’ expressed motivations for further education may relate to professional participation and clinical experience. Given that specialist nursing students are already practicing nurses whose educational choices are made within ongoing professional participation, a perspective that explicitly attends to practice, identity, and participation may be particularly relevant for interpreting how motivations for further education are expressed. Rather than conceptualizing motivation itself through either framework, the theories illuminate the professional and experiential contexts in which such motivations are articulated.

Situated Learning conceptualizes learning as participation in communities of practice, where knowledge, identity, and skills develop through engagement in socially and physically situated activities [26]. From this perspective, learning involves moving toward new forms of participation and shifting roles within professional practice, encompassing not only observable actions but also processes of meaning-making and identity negotiation [28]. This viewpoint has been widely used in studies of professional education, where learning is closely tied to practice [29]. Situated Learning thus offers a lens for examining how educational engagement relates to participation and development in professional practice [30]. In the present study, this perspective supports interpreting how motivations for further education are expressed in relation to participants’ ongoing involvement in clinical practice.

The From Novice to Expert framework complements this perspective by describing how nursing knowledge and competence may develop qualitatively through experience [27]. As practitioners gain experience, their performance shifts from greater reliance on rules and abstract principles toward increasingly context-sensitive forms of understanding [27]. Clinical experience can influence perception, judgment, and the ability to understand clinical situations holistically. Subsequent empirical research has highlighted the importance of experiential knowledge in how nurses interpret clinical situations and develop professionally [31]. In the present study, the framework is not used to classify participants by stages of competence or to equate length of clinical experience with expertise. Rather, it offers a perspective on how different amounts and forms of clinical experience may shape nurses’ perceptions of professional development and further education.

Together, these frameworks offered complementary interpretive perspectives for examining this study’s findings. Situated Learning emphasizes participation in professional practice and the social contexts in which learning occurs, while the From Novice to Expert framework highlights the potential significance of experiential knowledge in individual professional development.

### Aim

The aim of this study was to explore how nurses with different lengths of clinical experience articulate their motivations for specialist nursing education. The results may contribute to a more nuanced understanding of how professional experience shapes what nurses seek from further education, including their orientations towards learning, professional development, and other expected outcomes.

## Methods

### Design and context

Data in this study are part of a larger survey examining how clinical experience may affect self-efficacy, nursing competence, academic performance, and study outcomes among specialist nursing students at a higher education institution in Stockholm, Sweden.

In Sweden, specialist nursing education is a second-cycle professional program normally comprising 60 higher education credits. Midwifery education is a related postgraduate professional program comprising 90 credits. Both build on previous nursing education and professional registration and combine theoretical studies, clinical education, and an independent degree project. The programs in this study included both full-time over 3 terms (midwife) and part-time over 4 terms (all other programs), and most students combined their studies with ongoing clinical employment during the educational period.

This paper analyzes students’ reported motivation to pursue further education, based on qualitative free-text responses. The open-ended question was included in the original survey to capture participants’ own descriptions of their motivations for pursuing specialist nursing education. The possibility of conducting a qualitative analysis of these responses was considered during survey planning, and ethical approval covered both quantitative and qualitative analyses of the collected material. The research question was developed before analyzing the free-text responses, and an inductive analytical approach was selected to explore participants’ expressed motivations.

The study is reported in accordance with the Standards for Reporting Qualitative Research (SRQR) [32]. An SRQR checklist is provided as Supplementary Material.

### Participants and data collection

All students enrolled in one of six specialist nursing programs were invited to complete an online survey. The programs included Emergency Care, Prehospital Care, Cardiology, Cognitive Disease, Midwifery, and Palliative Care. Of the 213 students who received the survey, 94 responded, yielding a 44% response rate.

Students received an email invitation from a researcher without a teaching role or formal connection to their education to minimize potential dependency or perceived pressure to participate. The invitation was sent on 15 September 2025, and the survey ended on 26 October 2025. Participation was voluntary, and informed consent was obtained before accessing the survey. All participants were assured of confidentiality, and data were managed to prevent the identification of individual respondents.

The survey comprised three validated self-report instruments, demographic questions, and one open-ended question: “What is your motivation for pursuing specialist nursing education?” In the present study, basic demographic variables (age, gender, years of professional experience, and specialist nursing program) and responses to the open-ended question were analyzed. Data from the self-report instruments were not included. The open-ended question was mandatory, ensuring that all respondents provided a written response. The dataset consisted of 94 free-text responses describing students’ motivations for pursuing specialist nursing education. Responses ranged from 1 to 87 words, with a mean length of approximately 18 words.

Although the responses were generally brief, they were sufficient to identify recurring patterns in participants’ stated motivations. The aim of the analysis was not to generate richly detailed accounts of individual experiences but to explore how motivations for specialist nursing education were expressed across the sample.

### Data analysis

The study used an inductive, descriptive thematic analysis guided by Braun and Clarke’s six-phase approach [33]. The study then used cross-group comparisons and descriptive frequency counts of codes to identify possible patterns related to years of clinical experience [34]. This approach was considered appropriate because the study aimed to identify recurring patterns in brief free-text responses while remaining close to participants’ own descriptions.

The thematic analysis [33] began with multiple readings of all responses to become familiar with the dataset. The authors then conducted open coding across the material, focusing on clear descriptions of motives, perceived needs, goals, and factors motivating further education. Because the unit of analysis was limited, the authors kept codes close to participants’ wording to preserve semantic content.

The first author led the analysis in close collaboration with the second author. They independently coded the dataset and then compared their coding. Through discussion, the authors developed a shared interpretation of the material. Codes were then compared and grouped into initial themes based on shared meaning. At this stage, the third author joined the analysis process. The third author did not independently code the dataset but reviewed theme development, challenged interpretations, and contributed to discussions regarding theme structure and naming. The thematic analysis used no predefined theoretical categories [33].

All authors then reviewed themes against both the coded extracts and the full dataset to ensure internal consistency and clear distinctions among themes. The authors defined the final themes to reflect their central organizing meaning and illustrated them with representative quotes from the data. In this process, the authors translated quotations from Swedish into English. Particular attention was paid to preserving the meaning and intent of participants’ statements rather than producing literal word-for-word translations. Translated quotations were reviewed by the research team to ensure consistency and reliability with the original text.

Grouping participants by years of clinical experience enabled exploration of possible variation in motivations across nurses with differing professional backgrounds. These groupings were not intended to represent Benner’s [27] developmental stages or levels of competence but were used descriptively to examine whether motivations appeared to differ across nurses with varying lengths of clinical experience. The quantitative analysis counted the frequency of specific codes within each work-experience group (e.g., increased knowledge, professional growth, higher salary, greater responsibilities). Individual responses could be assigned to more than one code when participants expressed multiple motivations. Reported frequencies therefore represent the number of participants whose responses contained a particular code rather than the total number of responses [34].

The Novice to Expert framework [27] and Situated Learning [26] were used during the interpretation and discussion stages to support understanding of how expressed motivations might relate to professional identity formation, participation in clinical practice, and the development of experiential competence.

### Trustworthiness

Several strategies were applied to enhance the trustworthiness of this study. Credibility was strengthened through repeated data reviews and coding that stayed close to participants’ words, ensuring interpretations accurately reflected their responses. Two researchers independently coded the data, then compared their interpretations and discussed differences to develop a shared interpretation. The third author critically reviewed this process. Including illustrative quotations in the results further supports the connection between the data and the findings [35].

The research team comprised three nurse researchers and educators with experience in undergraduate and postgraduate nursing education and qualitative research. None of the authors taught in the specialist nursing program at this higher education institution at the time of data collection. Throughout the analysis, the authors reflected on how their professional backgrounds and assumptions could influence interpretation. The team held regular discussions to challenge interpretations and strengthen analytical reflexivity.

The team ensured dependability through a transparent, systematic analytic process in which they documented coding and theme development and maintained an audit trail of analytic decisions. At the theme development stage, a third researcher contributed to discussions about theme structure and naming, thereby strengthening the stability of the analysis [36]. Additionally, confirmability was reinforced by grounding interpretations in participants’ statements and by researcher triangulation [35]. The inductive approach to analysis, with theoretical frameworks introduced only at the interpretive stage, further prevented the influence of preconceived assumptions.

The study addressed transferability by clearly describing the study context, participants, and educational setting [36], enabling readers to evaluate the findings’ applicability to other postgraduate nursing education contexts. However, although participants represented several specialist nursing programs and a wide range of clinical experience, the 44% response rate should be considered when assessing the findings’ transferability, as non-response bias cannot be ruled out.

## Results

### Participants

The results include 94 specialist nursing students, aged 24 to 56 years (average age 37 years). They represented six specialist nursing programs, with most participants enrolled in the palliative care program. Table 1 shows the distribution of participants by educational program and years of clinical experience.

**Table 1:** Distribution of participants across educational programs and years of clinical experience

		Clinical experience		
	Total (*n* = 94)	≤ 2 years (*n* = 26)	3–10 years (*n* = 43)	≥ 11 years (*n* = 25)
Age in years, *mean (SD)*	37 (8)	33 (7)	36 (8)	43 (9)
Gender, *n (%)* Men Women Other/No answer	10 (10.6) 82 (87.3) 2 (2.1)	3 (11.5) 21 (80.8) 2 (7.7)	4 (9.3) 39 (90.7) 0	3 (12.0) 22 (88.0) 0
Educational program, *n (%)* Emergency care Prehospital care Cardiology Cognitive disorders Midwife Palliative Care	13 (13.8) 15 (16.0) 12 (12.8) 13 (13.8) 15 (16.0) 26 (27.6)	3 (11.5) 6 (23.1) 1 (3.8) 4 (15.4) 8 (30.8) 4 (15.4)	10 (23.3) 8 (18.6) 4 (9.3) 5 (11.6) 5 (11.6) 11 (25.6)	0 1 (4.0) 7 (28.0) 4 (16.0) 2 (8.0) 11 (44.0)

### Thematic analysis

The analysis identified four overarching motivational themes: Professional Role Advancement, Expanded Participation, Contributing to the Improvement of Clinical Practice, and Personal Advantages. Together, these themes describe how specialist nursing students position further education in relation to their professional practice, future trajectories, and life circumstances. Analytically, motivation may be understood here not only as intention but also as an expression of students’ positioning with respect to learning, knowledge, and participation in professional practice.

Participants were grouped by prior clinical experience. In the results, nurses with ≤ 2 years of experience were referred to as early-career, those with 3–10 years as mid-career, and those with ≥ 11 years as late-career. These categories served as an analytical framework to examine whether and how differences in years of clinical experience were associated with different orientations toward education. Rather than representing discrete stages of professional development, the categories provided a way to examine patterns in participants’ responses in relation to their prior clinical experience. Table 2 illustrates these two aspects.

**Table 2:** Thematic analysis and quantification of codes depending on clinical experience

Code	Clinical experience in years			Subtheme	Theme
	**≤ 2** *(n* = 26)	**3–10** *(n* = 43)	**≥ 11** *(n* = 25)		
Learn more	3	9	2	Increased knowledge	**Professional Role Advancement**
Gain more knowledge	4	12	5		
Deepen my knowledge	3	9	13		
Increase competence	2	4	3	Better ability to act	
Gain tools	-	2	-		
Development as a nurse	2	10	6		
Anchor knowledge	1	-	-		
Status	-	1	-	Enhanced professional agency	
Gain more responsibility	1	2	-		
Work independently	-	1	1		
Security	4	2	-	Increased confidence	
Exciting and scary	2	-	-		
Interest	9	1	1	Desire for a specific professional role	**Expanded Participation**
A dream	7	1	-		
I see myself in this role	2	-	-		
Want to become a midwife	5	4	-		
I want to become a specialist nurse	1	1	2		
Had a plan from the beginning	3	1	.		
I want to work in a specific area	2	1	1		
I want to pursue further studies	4	-	-	Academic participation	
I want to conduct research	-	-	2		
Improvement for a specific group of patients	-	3	1	Vision for change	**Contributing to the Improvement of Clinical Practice**
Share knowledge in other contexts	1	-	-		
Expand professional network	-	1	-		
Development work	-	6	3	Organizational development	
Develop competence within the organization	-	4	3		
Increase colleagues’ competence.	-	4	3		
Inspire new ways of working	-	-	2		
Individualized care	-	-	1	Patient-centered care	
Improve care for my patients	4	3	2		
Increase salary	1	10	1	Economic advantages	**Personal Advantages**
Influence salary development	-	2	1		
Position/employment	-	2	1	Work-related conditions	
Working hours	1	2	-		
Support from colleagues	-	1	-		
Change in life	-	2	-	Increased well-being	
Improved situation	-	2	1		
Want more meaning	-	-	2		
Make healthier priorities	1	-	-		
Have fun	1	-	2		

#### Professional role advancement

Professional role advancement was a pervasive motivational orientation among participants. It involved a desire for increased knowledge and confidence, a greater ability to act in clinical settings, and enhanced professional agency. Participants frequently expressed this directly, for example, “*To gain more knowledge*” and “*I want to learn more*”. Across the responses, learning was commonly described as something expected to strengthen participants’ professional practice. However, participants with different lengths of clinical experience appeared to emphasize somewhat different aspects of professional role advancement.

Early-career nurses often expressed a desire to “*learn more*,” “*gain knowledge*,” and feel more secure in their professional roles. Some described their motivation as wanting to “*Extend my knowledge in an area that I am most interested in*” or stated that “*I believe that more knowledge and understanding provide a more secure foundation for working as a nurse.”* In these responses, participants primarily described further education as a way to acquire knowledge and strengthen confidence in clinical practice.

Mid-career nurses described further education as an opportunity to gain knowledge to better understand clinical situations. One participant described this as wanting “*to gain broader knowledge and deeper understanding for complex situations*.” Compared with the other groups, these responses more often emphasized the relationship between theoretical knowledge and clinical experience for professional development. One participant expressed:

To deepen my knowledge and to be able to place the increasingly demanding and stressful everyday life we lead within a theoretical context.

Late-career nurses framed professional advancement in terms of specialization, deepening knowledge, or strengthening their position in areas where they already had substantial experience. Education was described as an opportunity to further develop knowledge in an established area of practice and, in some cases, to obtain formal qualifications that were perceived as valuable. Two participants expressed this as:

Being able to delve deeper into the type of healthcare I have been working in for so long now.

Despite extensive clinical experience and courses studied in cardiology, a specialist nursing education carries more weight.

Thus, although participants across all experience groups expressed motivation for professional advancement, the emphasis differed in the descriptive coding. Early-career nurses more often emphasized acquiring knowledge and confidence, mid-career nurses broadening and integrating knowledge in relation to clinical practice, and late-career nurses deepening or formalizing knowledge within established areas of experience. Although these groups should not be viewed as a classification based on nursing expertise, these patterns suggest differences in what participants expected further education to provide in their professional advancement based on their previous clinical experience.

#### Expanded participation

Expanded participation captured motivations related to pursuing desired professional roles, entering new areas of practice, or broadening opportunities for professional and academic engagement. Across the responses, further education was frequently described as a pathway to a different role or to new professional opportunities.

Several early-career nurses described future-oriented aspirations, referring to desired roles, long-held ambitions, or positions they hoped to attain. Further education was therefore commonly presented as a way to access roles participants had not yet entered. One participant stated:

This was the reason I started the nursing program in the first place.

Some participants also described these anticipated role transitions as both desirable and challenging. For example:

I always wanted to be a midwife, and when I got in, I just wanted to go for it, even though it is a little scary!

Mid-career nurses more frequently described further education as enabling changes within their existing professional practice. Their responses emphasized both expanding their scope of practice within a familiar area and into new fields. In these responses, further education was associated with developing one’s role and responsibilities. For example, two participants stated:

I want to grow professionally in a field that is very close to my heart. My interest in this field has gradually developed over the years.

I plan to work in an emergency room or on an ambulance as soon as I finish my education. The reason I’m working at a nursing home right now is so I can support myself and my family while I’m in school.

Among late-career participants, expanded participation involved opportunities to move into new professional contexts or activities beyond their current clinical role. Examples included engaging in development work, quality improvement, education, or research. One participant described this as:

[I want to] gain higher-level skills and be able to work with improvements and changes. Also, be able to find a path into research as a long-term goal.

Across the experience groups, motivations related to expanded participation therefore appeared to differ in emphasis. Early-career participants frequently described aspirations to enter desired roles and were more heavily represented in this category compared to the other groups, which may suggest that specialist nursing education can be viewed as a stepping stone toward their higher goal. Meanwhile, mid-career participants emphasized developing responsibilities within professional practice, and late-career participants mostly referred to opportunities to extend their professional role into new professional or academic domains, illustrating the breadth of expectations for the professional impact education is expected to have on the individual nursing role.

#### Contributing to the Improvement of clinical practice

This theme captured motivations related to improving clinical practice, including providing better patient care, supporting colleagues, contributing to improvement work, and influencing organizational development. Participants described further education as a way of strengthening their ability to contribute to healthcare development. Across responses, participants described improvement in different ways, from improving care in individual patient encounters to contributing to workplace development and broader changes in healthcare practice.

It was observed that early-career nurses described improvement primarily in direct patient care and their ability to respond appropriately in clinical situations. Their responses focused on developing knowledge and competence that could improve the care they provide. Two participants expressed this as:

I want to assist and be present. Therefore, I would like to learn so that I can respond appropriately.

Improve knowledge about symptom management and providing the best possible care for patients with advanced cancer and their families.

Mid-career nurses more often mentioned knowledge sharing, supporting colleagues, and participating in improvement initiatives. Their responses suggested an interest in contributing to changes within their immediate workplace or organization. For example, one participant stated:

I want to become stronger and more confident in my profession, which is essential in specialized care for driving development initiatives and supporting colleagues on a general level.

Late-career nurses more often expressed ambitions to influence broader areas of practice or contribute to the development of healthcare services. Their responses included aspirations to shape care, initiate development work, or influence how particular areas of healthcare were organized. One participant responded:

I work in municipal healthcare at a residential facility that provides dementia care. I’m involved in efforts to advance palliative care at the primary care level. I’m continuing my education to gain deeper knowledge in this field and have a greater impact.

Thus, in this category, mid-career and late-career nurses were more represented than participants with less experience. Also, early-career nurses focused on improving their own clinical performance and patient care, whereas mid-career participants more often referred to influencing local practice and colleagues, and late-career participants more often described ambitions related to broader practice development. These statements illustrate the variety of contexts in which students participate and which they may expect to influence through specialist nursing education.

#### Personal advantages

Personal advantages formed a motivational theme related to the life conditions surrounding further education. Participants referred to factors such as salary, employment opportunities, work arrangements, flexibility, variation, and personal well-being. Personal advantages were therefore described as practical and individual circumstances that could motivate participation in further education alongside professional and practice-related motivations.

Economic motives were explicitly expressed, particularly among mid-career participants, with statements such as “*Salary increase is clearly motivating.”* Responses also included considerations related to work and family life. For example, one participant described further education as compatible with family circumstances, stating: “*This means I can be home more while the children are young*.”

Some participants also described further education as an opportunity for variation, enjoyment, or renewal in their working lives. One mid-career participant explained:

It is also fun to have a bit of a change and do something completely different from just working. In the long run, it is also an advantage to be a specialist nurse, not only for the knowledge, but also because it is easier to get certain jobs and it gives an extra boost to your salary.

Notably, salary and employment-related motivations appeared considerably more often in responses from mid-career participants than among early- and late-career participants, which may reflect different stages of career progression as well as different stages in personal life. Nevertheless, the breadth of personal motivational factors shows that students are sometimes driven not by professional goals, but by motivations outside both the educational and professional context.

## Discussion

The findings suggest that specialist nursing students’ motivations for further education may be linked to their understanding of their professional roles, prior clinical experience, and future opportunities in practice. They also indicate that students may differ in what they expect further education to provide, both in terms of knowledge and learning and in its anticipated impact on their professional and personal lives. This is relevant for educators and higher education institutions seeking to understand the diverse backgrounds students bring to specialist nursing education. The findings align with earlier studies of specialist and postgraduate nursing students, in which motivation has been described in relation to professional development, role change, and clinical responsibility [7, 23]. This study adds to that knowledge by illustrating how motivational orientations and expectations for further education differed among students with varying lengths of prior clinical experience.

The descriptive patterns observed across the experience groups suggest that early-career participants more often emphasized acquiring knowledge, confidence, and aspiring roles; mid-career participants emphasized broadening knowledge and were largely motivated by personal concerns, whereas late-career participants emphasized deepening knowledge or pursuing formal qualifications within established areas of practice. These expectations appeared to concern not only knowledge acquisition but also how education was expected to influence participants’ future participation in the professional community, responsibilities, and opportunities.

Although the grouping in this study was not a proxy for nursing competence, Benner’s novice-to-expert framework [27] may still offer a theoretical lens for interpreting these differences. The framework highlights how experience shapes practitioners’ understanding and use of knowledge in clinical settings. From this perspective, the greater emphasis on knowledge acquisition and security among participants with shorter clinical experience may reflect the importance of formal knowledge and rules when experience is still limited. Similarly, the emphasis among participants with longer clinical experience on deepening, formalizing, or extending existing knowledge may reflect accumulated experiential knowledge. Together, these patterns suggest that clinical experience may influence not only how much knowledge students bring to specialist education, but also how they perceive the role and value of formal knowledge.

From a pedagogical perspective, these findings may indicate a need to recognize that students entering specialist nursing education do not necessarily share the same relationships with knowledge and learning and may have different needs for how new knowledge is introduced, contextualized, and connected to practice. Rather than suggesting different curricula for predefined developmental stages, the findings suggest that educational design could benefit from flexibility and responsiveness to students’ varying experiential backgrounds. Students with relatively limited clinical experience might benefit from explicit expectations and structured opportunities to connect theoretical knowledge to clinical situations [31]. Students with more extensive experience may, in turn, value opportunities to critically examine established practice, articulate experiential knowledge, or apply theoretical perspectives to complex professional problems [37].

In addition, participants frequently expressed their motivations in relation to professional roles, clinical practice, and opportunities to influence healthcare. They also described expectations about how education could affect their professional position, future possibilities, and everyday working life, illustrating the breadth of expectations for the professional impact education is expected to have at the individual level. This supports the relevance of considering motivation in relation to professional and educational contexts rather than solely as an individual psychological characteristic [10]. Earlier research has similarly shown that prior clinical experience can influence how students perceive and use higher education [38]. However, it has been pointed out that motivation is likely not a fixed factor [25], and the present study cannot determine whether or how motivational differences develop over time or influence academic engagement, persistence, or educational outcomes.

When interpreted through Situated Learning [26], the findings may suggest that students understand further education, in part, as tied to participation in professional practice. Across themes, students often described education as a way to strengthen their ability to act in clinical settings, enter desired roles, broaden professional opportunities, or contribute to the professional community. The findings illustrate that students come from a variety of professional and personal contexts and may expect specialist nursing education to offer opportunities to influence those contexts through their future practice. From this perspective, learning relevance to practice may be essential to their motivation for further education. This interpretation aligns with research indicating that professional relevance supports motivation in nursing education [17, 19].

The findings may therefore have implications for how academic and clinical learning are connected in specialist nursing education. Educational approaches that explicitly link theoretical content to clinical contexts, such as case-based learning, practice-linked assignments, or workplace-based projects, may help students connect academic learning to issues they encounter or anticipate in professional practice [39]. For students with diverse clinical backgrounds, such approaches may also allow prior experiences to serve as a resource for learning rather than merely a characteristic of the learner. Importantly, connecting education to authentic practice may also help accommodate students whose expectations extend beyond knowledge acquisition to include broader aspirations to change or influence their professional context [40].

In this light, collaboration between higher education institutions and clinical workplaces could offer opportunities to engage with authentic professional problems and to connect academic learning with real-life healthcare development. Similar motivations to improve practice have been identified in postgraduate nursing education internationally [22–24]. Strong partnerships between higher education institutions and clinical settings may support meaning-making, relevant learning experiences and opportunities for advanced clinical reflection [41]. However, previous research has shown a discrepancy between what nurses expected and what was actually possible after they completed their specialist education [23]. This presents an opportunity for employers in clinical settings to increase motivation for further education by creating opportunities for specialist nurses to contribute to healthcare development and improvement, both within their own workplace and through networks with other organizations.

The findings also have potential implications for educators’ roles. If students enter specialist nursing education with varied clinical experiences and learning expectations, differences in engagement should not be assumed to reflect a lack of motivation or commitment. Instead, educators may need to consider how students’ prior experiences shape the questions they ask, the knowledge they seek, the relevance they ascribe to educational activities, and the support they need to grasp the course content. Autonomy-supportive teaching has been linked to higher intrinsic motivation and effort [20], and approaches that acknowledge students’ experiences while offering opportunities for independent choice, responsibility, and professional relevance may therefore be valuable. Furthermore, it has been pointed out that nurses undergoing specialist education and clinical practice often expect in-depth knowledge following specialist nursing education, and that the training is sometimes perceived as too general, which reduces its perceived value and, ultimately, the motivation to invest the time and effort required to complete it [7]. Here, of course, higher education institutions have a responsibility to offer education relevant to the realities of clinical practice.

At the same time, in line with earlier research [22, 24], this study found that motivation was not always primarily educational or professional. This extends previous descriptions of motivation by suggesting that participation in specialist nursing education may also be embedded in broader life situations, where education is valued for reasons that are not necessarily related to acquiring knowledge or advancing professionally. Such motivations do not imply a lack of commitment to education; rather, they highlight that students may enter the same program with fundamentally different expectations of what participation could provide.

Nonetheless, educational flexibility should not mean assigning students to predetermined learning pathways based solely on years of experience. Length of clinical experience is one aspect of a student’s professional background, and individuals with similar experience may differ substantially in competence, prior roles, educational experiences, and learning needs. A potentially useful pedagogical approach may therefore be to create learning environments where diverse experiences are actively used as resources while maintaining shared academic and professional learning goals, with additional support for students with less clinical experience [9]. One strategy could be to offer foundational course components to students with less experience, ensuring all students share a common knowledge base they can apply in more advanced learning activities.

In addition, recognizing the diversity of students’ motivations may help educators avoid assuming that all students are primarily oriented toward the same educational or professional outcomes. Educators should also consider contextual factors such as workload, family circumstances, and employment conditions, as these may shape students’ opportunities to succeed in further education [24]. Together with the present findings, these contextual considerations suggest that motivation for specialist nursing education is situated not only within educational and professional trajectories but also within the broader circumstances of students’ lives.

### Strengths and limitations

This study has several strengths and limitations. The study included 94 participants from six specialist nursing programs, providing variation in professional and educational contexts. The open-ended survey question also allowed participants to express their motivations in their own words rather than selecting from predefined response alternatives. This provided breadth across a relatively large number of participants within the study context. However, the qualitative depth was limited. The study was based on responses to a single question, and individual responses were often brief. Consequently, the data did not provide the depth that interviews or focus groups would have done, and the findings should be interpreted as descriptive patterns in brief accounts rather than comprehensive explanations of participants’ motivations. The mandatory nature of the question may also have influenced response length and depth, as participants had to write something, but one word was enough.

The analysis used an inductive, descriptive thematic approach informed by Braun and Clarke’s six-phase framework [33], complemented by descriptive frequency counts. As with all qualitative analysis, the researchers’ perspectives and theoretical interests influenced interpretation. The involvement of a third researcher provided an opportunity for critical discussion of the developing analysis; however, this should not be understood as establishing an objective or independent verification of the findings. Furthermore, the translation from Swedish to English involves an interpretive step that may have affected how the participants’ messages were conveyed.

Comparing groups by years of clinical experience allowed exploration of variation in descriptive coding, but these groups should not be understood as representing empirically established stages of professional development. Years of experience do not directly indicate competence or expertise [27], and the cross-sectional design cannot demonstrate developmental change over time. Furthermore, the frequency counts were descriptive and were not intended to demonstrate statistically significant differences between groups.

In addition, the study was conducted at a single institution, and 56% of invited students did not participate. Non-response bias is therefore possible, and participants may not represent the motivations of specialist nursing students in general. Instead, they offer an exploratory account of how motivations for further education were expressed within this sample and how these expressions varied descriptively with length of clinical experience.

## Conclusion

The findings suggest that length of prior clinical experience may shape not only what students know but also what they seek from formal education and how they engage with new knowledge. Specialist nursing students enter further education with diverse motivations and expectations, shaped by their previous clinical experience and broader professional and personal circumstances. Consistent with perspectives on situated learning and the development of nursing expertise, the findings suggest that students’ prior experiences and positions in clinical practice may influence how they approach and make sense of further education. For educators, this underscores the importance of not assuming that knowledge acquisition and professional development are the primary motivation for all students. Rather, education may serve different purposes for different students, and recognizing these varied orientations may help shape educational environments that respond to students’ different starting points and expectations while maintaining shared academic and professional goals.

However, this study could not explore how motivation may vary over the course of the program. Future research could examine these patterns using longitudinal designs to explore how motivations and relationships with learning change over time. Interview-based studies could also provide greater depth regarding how nurses with different professional backgrounds understand their motivations for specialist education. Studies across multiple institutions and educational contexts would further help determine how transferable the patterns identified here are to other settings.

## Supplementary Information

Below is the link to the electronic supplementary material.

Supplementary Material 1

## Data Availability

The datasets generated and/or analyzed during the current study are not publicly available due to ethical reasons, but are available from the corresponding author on reasonable request.

## References

[1] Notarnicola I, Dervishi A, Duka B, Grosha E, Gioiello G, Carrodano S, et al. A systematic review of nursing competencies: addressing the challenges of evolving healthcare systems and demographic changes. Nurs Rep. 2025;15(2):56. 10.3390/nursrep15020056 PMC11858704 39997792

[2] Bryant-Lukosius D, Valaitis R, Martin-Misener R, Donald F, Peña LM, Brousseau L. Advanced Practice Nursing: A Strategy for Achieving Universal Health Coverage and Universal Access to Health. Rev Lat Am Enfermagem. 2017;25:e2826. 10.1590/1518-8345.1677.2826 PMC5288863 28146177

[3] Woo BFY, Lee JXY, Tam WWS. The impact of the advanced practice nursing role on quality of care, clinical outcomes, patient satisfaction, and cost in the emergency and critical care settings: a systematic review. Hum Resour Health. 2017;15(1):63. 10.1186/s12960-017-0237-9 PMC5594520 28893270

[4] Zhang X, Wang F, Wang Q, Liu H, Lee S-Y. The link between patient safety competence and adverse event among master of nursing students: a cross-sectional mixed-methods study. BMC Nurs. 2024;23(1):539. 10.1186/s12912-024-02213-3 PMC11304561 39112994

[5] Jokiniemi K, Miettinen M. Specialist nurses’ role domains and competencies in specialised medical healthcare: a qualitative descriptive study. Int J Caring Sci. 2020;13(1):171–9.

[6] Ares TL. Role Transition After Clinical Nurse Specialist Education. Clin Nurse Specialist. 2018;32(2):71–80. 10.1097/NUR.0000000000000357 29419579

[7] Tiliander A, Olsson C, Kalèn S, Ponzer S, Fagerdahl A. Exploring career choices of specialist nurse students: Their decision-making motives. A qualitative study. Nurs Open. 2024;11(7):e2241. 10.1002/nop2.2241 PMC11259742 39032132

[8] Mao A, Lu S-e, Lin Y, He M. A scoping review on the influencing factors and development process of professional identity among nursing students and nurses. J Prof Nurs. 2021;37(2):391–8. 10.1016/j.profnurs.2020.04.018 33867096

[9] Chambers A, Whitfield C. Factors influencing postgraduate nursing students’ engagement with online learning in higher education: A mixed methods literature review. Nurse Educ Pract. 2025;88:104570. 10.1016/j.nepr.2025.104570 41005049

[10] İncesu O, Sönmez B. The Relationship Between Attitudes Toward Postgraduate Education, Academic Self-Efficacy and Academic Motivation of Nurses Pursuing Postgraduate Education: A Cross-Sectional Study. Turkiye Klinikleri J Nurs Sci. 2024;16(4):1121–31.

[11] Jokiniemi K, Pietilä AM, Kylmä J, Haatainen K. Advanced nursing roles: a systematic review. Nurs Health Sci. 2012;14(3):421–31. 10.1111/j.1442-2018.2012.00704.x 22950621

[12] Cook DA, Artino AR Jr. Motivation to learn: an overview of contemporary theories. Med Educ. 2016;50(10):997–1014. 10.1111/medu.13074 PMC5113774 27628718

[13] Stavropoulou G, Daniilidou A, Nerantzaki K. Exploring the Interplay of Motivation, Self-Efficacy, Critical Thinking, and Self-Regulation in Predicting Academic Achievement Among University Students. F1000Research. 2025;14:344. 10.12688/f1000research.161821.2 PMC12345619 40808874

[14] Amadi WA, Du Plessis M, Balogun TV. A systematic review of the factors influencing the motivation to lead among students in higher education. Front Educ.2025;10:1564445.

[15] Törmänen T, Ketonen E, Lehtoaho E, Turunen M, Zabolotna K, Shubina T, et al. Situational Motivation in Academic Learning: A Systematic Review. Educational Psychol Rev. 2025;37(2):56.

[16] Sušilović I, Ljubičić M, Matijaš T, Bokan I, Marendić M. Motivations and attitudes toward further education: a cross-sectional, Descriptive Predictive Study. Nurs Rep. 2025;15(6):190. 10.3390/nursrep15060190 PMC12195818 40559481

[17] Hamm J, Yeh P-M. Factors affecting academic motivation in undergraduate nursing students: A scoping review. Nurse Educ Pract. 2024;80:104135. 10.1016/j.nepr.2024.104135 39270487

[18] Nilsson KE, Warrén Stomberg MI. Nursing students motivation toward their studies - a survey study. BMC Nurs. 2008;7:6. 10.1186/1472-6955-7-6 PMC2386463 18439276

[19] Rafii F, Saeedi M, Parvizy S. Academic Motivation in Nursing Students: A Hybrid Concept Analysis. Iran J Nurs Midwifery Res. 2019;24(5):315–22. 10.4103/ijnmr.IJNMR_177_18 PMC6714123 31516515

[20] Torbergsen H, Utvær BK, Haugan G. Nursing students’ perceived autonomy-support by teachers affects their intrinsic motivation, study effort, and perceived learning outcomes. Learn Motiv. 2023;81:101856.

[21] Tiliander A, Olsson C, Kalèn S, Ponzer SS, Fagerdahl AM. Factors affecting nurses’ decision to undergo a specialist education and to choose a specialty. Nurs Open. 2023;10(1):252–63. 10.1002/nop2.1300 PMC9748047 35941100

[22] Zhu D, Zhu H, Xie W, Fu H, Lv J, Yuan Q, et al. Motivations and internship experiences of registered nurses pursuing Master of Nursing Specialist education in China: a phenomenological analysis. BMC Nurs. 2025;24(1):1418. 10.1186/s12912-025-04074-w PMC12636178 41267105

[23] Oldland E, Redley B, Botti M, Hutchinson M. Nurses motivations and desired learning outcomes of postgraduate critical care studies: A descriptive exploratory study. Australian Crit Care. 2023;36(4):586–94. 10.1016/j.aucc.2022.05.004 35732556

[24] Ingram LA, O’Kane CE, Mullay S. Unlocking the Drive: Exploring the Hidden Motivations and Challenges of Nurses Pursuing Postgraduate Education: A Narrative Review of the Literature. J Radiol Nurs. 2025;44(1):57–64.

[25] Bingen HM, Olaussen C, Rød I, Sundfær H, Solberg MT. Postgraduate nursing students’ motivation for learning: A mixed-method study exploring changes in academic motivation. Nurse Educ Today. 2025;154:106834. 10.1016/j.nedt.2025.106834 40749526

[26] Lave J, Wenger E. Situated learning: Legitimate peripheral participation. New York, NY, US: Cambridge University Press; 1991. pp. 138–p.

[27] Benner P. From novice to expert: Excellence and power in clinical nursing practice. Addison-Wesley; 1984.

[28] Handley K, Sturdy A, Fincham R, Clark T. Within and Beyond Communities of Practice: Making Sense of Learning Through Participation, Identity and Practice. J Manage Stud. 2006;43(3):641–53.

[29] Olmos-Vega FM, Stalmeijer RE. Using theoretical engagement to understand workplace learning across contexts-Bringing worlds apart together. Med Educ. 2025;59(1):65–74. 10.1111/medu.15481 PMC11662299 39104362

[30] Wenger E. Communities of practice: learning, meaning, and identity. Cambridge University Press; 1998.

[31] ten Hoeve Y, Kunnen S, Brouwer J, Roodbol PF. The voice of nurses: Novice nurses’ first experiences in a clinical setting. A longitudinal diary study. J Clin Nurs. 2018;27(7–8):e1612–26. 10.1111/jocn.14307 29446496

[32] O’Brien BC, Harris IB, Beckman TJ, Reed DA, Cook DA. Standards for reporting qualitative research: a synthesis of recommendations. Acad Med. 2014;89(9):1245–51. 10.1097/ACM.0000000000000388 24979285

[33] Braun V, Clarke V. Thematic analysis: a practical guide. Sage; 2022.

[34] Sandelowski M. Real qualitative researchers do not count: the use of numbers in qualitative research. Res Nurs Health. 2001;24(3):230–40. 10.1002/nur.1025 11526621

[35] Shenton AK. Strategies for ensuring trustworthiness in qualitative research projects. Educ Inform. 2004;22(2):63–75.

[36] Nowell LS, Norris JM, White DE, Moules NJ. Thematic Analysis:Striving to Meet the Trustworthiness Criteria. Int J Qualitative Methods. 2017;16(1):1609406917733847.

[37] Kohanová D, Šulajová V, Zrubcová D, Pavelová Ľ, Sollárová A. Postgraduate nursing students’ reflection on safe clinical practice: a descriptive qualitative study. Kontakt. 2025;27(3):252–8.

[38] Bennett D, Knight E, Li I. The impact of pre-entry work experience on university students’ perceived employability. J Furth High Educ. 2023;47(8):1140–54.

[39] Afonso C, Amaral O. From Theory to Practice: An Implementing Case-Based Learning in a Family Health Nursing Master’s Course. SAGE Open Nurs. 2025;11:23779608251379102. 10.1177/23779608251379102 PMC12575927 41179245

[40] Wang S, Yuan Y, Zhou C, He H, Wang Y. Postgraduate nursing students’ experiences in a project-based learning model: a qualitative focus group study. BMC Med Educ. 2026;26(1):330. 10.1186/s12909-026-08649-2 PMC12924622 41612312

[41] Larsson M, Sundler AJ, Blomberg K, Bisholt B. The clinical learning environment during clinical practice in postgraduate district nursing students’ education: A cross-sectional study. Nurs Open. 2023;10(2):879–88. 10.1002/nop2.1356 PMC9834198 36062832

[42] World Medical Association. WMA declaration of Helsinki: Ethical principles for medical research involving human subjects. 2013 [Available from: https://www.wma.net/what-we-do/medical-ethics/declaration-of-helsinki/. 10.1001/jama.2013.281053 24141714

